# Hybrid LTCC–Polyimide Approach for High-Sensitivity Mechanical Sensing Applications

**DOI:** 10.3390/s26051419

**Published:** 2026-02-24

**Authors:** Fares Tounsi, Nesrine Jaziri, Mahsa Kaltwasser, Michael Fischer, Denis Flandre, Jens Müller

**Affiliations:** 1 SMALL Group, ICTEAM Institute, Université Catholique de Louvain, 1348 Louvain-la-Neuve, Belgium; denis.flandre@uclouvain.be; 2 Electronics Technology Group, Institute of Micro and Nanotechnologies MacroNano, Technische Universität Ilmenau, 98693 Ilmenau, Germany; nesrine.jaziri@tu-ilmenau.de (N.J.); mahsa.kaltwasser@tu-ilmenau.de (M.K.); michael.fischer@tu-ilmenau.de (M.F.); jens.mueller@tu-ilmenau.de (J.M.)

**Keywords:** LTCC technology, Kapton^®^ substrate, tunable RF components, reflow soldering, low-temperature bonding, indium electroplating, high sensitivity sensors

## Abstract

Low-Temperature Co-Fired Ceramic (LTCC)-based mechanical sensors are inherently limited by the thickness and rigidity of multilayer ceramic stacks, which restrict miniaturization and mechanical compliance. To overcome these constraints, this work presents a hybrid LTCC/Kapton^®^ platform enabling high-sensitivity mechanical sensing through mechanically tunable RF passive components. The proposed approach integrates a flexible polyimide membrane, bonded onto an LTCC substrate at low temperatures using selectively electroplated indium pillars that simultaneously define the air gap and provide mechanical fixation. Inductance tuning is achieved via metal-shielding proximity effects, whereas capacitance tuning relies on force-controlled air-gap modulation in a metal–insulator–metal configuration. The fabrication process ensures precise gap control, high compliance, and structural robustness without requiring deformable ceramic membranes. Experimental characterization, including three-dimensional surface profiling and impedance measurements, demonstrates a 48% inductance tuning range with a sensitivity of 0.715 nH/mN and a 36% capacitance tuning range with a sensitivity of 47.3 fF/mN at 1 MHz. The proposed hybrid platform provides a compact and scalable solution for high-sensitivity sensors and mechanically reconfigurable RF components suitable for harsh-environment and adaptive electronics applications.

## 1. Introduction

Mechanical sensing, including pressure, force, and tactile detection, is playing an increasingly critical role in harsh and high-frequency environments across aerospace, automotive, energy, and industrial systems [1,2]. Conventional mechanical sensors are predominantly based on micromachined silicon technologies; however, their performance is severely limited in harsh environments due to silicon degradation at elevated temperatures, high humidity, and aggressive chemical conditions, as well as leakage current issues above ~150 °C [3,4,5]. In particular, micromachined silicon pressure sensors relying on p–n junctions, MOS structures, or piezoresistive elements formed in doped silicon exhibit strong temperature-dependent leakage currents at high temperatures, leading to signal drift, increased noise, and ultimately device failure [6].

In contrast, Low-Temperature Co-Fired Ceramic (LTCC) technology has emerged as a promising technology for sensor and RF system integration, offering reliable operation under high-temperature, high-pressure, and chemically aggressive conditions. This robustness arises from the intrinsic thermal and mechanical strength of ceramic materials (rupture strength ≈ 320 MPa, Young’s modulus ≈ 120 GPa), their excellent chemical stability, as well as LTCC’s superior electrical insulation, low dielectric loss, and compatibility with multilayer fabrication processes [7]. Furthermore, LTCC allows the monolithic integration of embedded passive components, including inductors, capacitors, resistors, vias, and cavities, within a single ceramic substrate using additive manufacturing techniques [8]. As a result, LTCC has been widely adopted for RF and microwave circuits, as well as for sensors measuring pressure, temperature, flow, and gas concentration in harsh environments [9,10].

Numerous LTCC-based pressure and mechanical sensors have been reported in the literature. Fournier et al. [11] developed an integrated LTCC multi-sensor for industrial compressed air diagnostics that combines pressure, flow, and temperature sensing with embedded signal-conditioning electronics in a single ceramic package. Lin et al. [12] proposed a passive wireless LTCC-based LC resonant pressure and temperature sensor operating over pressure ranges of 140–850 kPa and temperatures up to 500 °C. More recently, passive wireless LTCC sensors have been demonstrated for harsh-environment monitoring of pressure, temperature, and gas concentration using LC resonance and inductive coupling techniques [13,14]. These studies clearly demonstrate LTCC’s suitability as a robust platform for integrated sensing systems that combine mechanical transduction and RF signal processing.

Despite these advances, the fabrication of deformable microstructures within LTCC substrates remains a significant challenge [15]. The lamination and co-firing steps impose strong mechanical constraints that hinder the realization of thin membranes, narrow air gaps (<50 µm), and compliant three-dimensional structures without deformation, warpage, or collapse [16,17]. These limitations become increasingly pronounced as the ceramic layer thickness decreases. Consequently, LTCC-based pressure sensors typically exhibit limited sensitivity compared with silicon-based counterparts, and many tunable or deformable elements are implemented as external components rather than being fully co-integrated within the ceramic substrate [13,18]. In parallel, polymer-based flexible sensors, particularly capacitive pressure sensors employing polyimide (PI, commercially known as Kapton^®^) membranes, have attracted considerable attention due to their high mechanical compliance, fast response, and ease of fabrication [19,20]. However, purely polymer-based sensors often suffer from limited long-term stability and environmental sensitivity [21], while mechanical mismatch can cause deformation and electrical degradation due to electrode delamination, cracking, or conductivity changes under stress [22]. These drawbacks significantly restrict their applicability in high-temperature, high-pressure, or chemically aggressive environments, where ceramic-based technologies such as LTCC are more suitable.

To overcome the respective limitations of ceramic and polymer technologies, this work proposes a hybrid integration approach that combines flexible polymer membranes with LTCC substrates. Such hybrid systems have the potential to merge the high mechanical compliance of PI membranes with the robustness, hermeticity, and RF performance of LTCC. Nevertheless, the reliable co-integration of flexible polymer membranes with LTCC substrates, while ensuring precise air-gap control, robust bonding, and compatibility with standard LTCC processing, remains a major challenge. In this paper, we introduce a novel LTCC/Kapton^®^ hybrid approach that enables high-sensitivity mechanical sensing through mechanically tunable RF inductors and capacitors. While ceramic–polymer hybrid systems have been reported for structural reinforcement, dielectric composites, or protective encapsulation [23], these approaches employ polymers in passive roles and does not exploit its mechanical compliance for active electromechanical transduction. The proposed concept is based on the co-integration of a flexible, copper-clad PI membrane with an LTCC substrate embedding planar inductive and capacitive structures. Mechanical loading induces controlled membrane deflection, which alters the physical field interactions governing the passive components without requiring deformable ceramic membranes or buried cavities. Inductance tuning is achieved via metal-shielding proximity effects, where the approach of a grounded conductive plate modifies the magnetic field distribution of an LTCC-integrated spiral inductor via eddy-current interactions. In contrast, capacitance tuning relies on electrostatic modulation of a precisely defined air gap between the movable membrane electrode and an LTCC-embedded electrode. By externalizing mechanical compliance to the PI membrane, the proposed architecture eliminates the need for buried ceramic cavities, thin ceramic membranes, or sacrificial layers, which are known to introduce warpage, collapse, and yield limitations during LTCC lamination and co-firing. The fabrication strategy is compatible with standard LTCC processing and relies on low-temperature bonding techniques, allowing seamless co-integration with embedded passives, vias, cavities, and RF interconnects on a single ceramic substrate. Beyond mechanical sensing, this hybrid platform provides a generic route toward mechanically reconfigurable inductors and capacitors, which are key elements in adaptive RF front-end architectures, including tunable matching networks, filters, oscillators, and phase shifters, and reconfigurable RF front-end circuits [24].

This paper is organized as follows. Section 2 presents the basic concept and operating principles of the mechanically tunable inductive and capacitive elements. Section 3 details the fabrication process of the hybrid LTCC/Kapton^®^ platform. Section 4 discusses the modeling and simulation results, while Section 5 presents the experimental characterization and tuning performance. Finally, Section 6 concludes the paper with key results and perspectives.

## 2. Basic Concept and Principles

Polyimide (PI) is a widely used, low-cost substrate for flexible electronics owing to its favorable dielectric properties, excellent thermal stability, and chemical resistance [25]. Mechanically, polyimide is highly compliant under bending and twisting while remaining essentially non-stretchable and non-compressible [26,27]. With a Young’s modulus of ~2.5 GPa, nearly 40 times lower than that of LTCC, PI provides significantly greater mechanical flexibility. In addition, PI films can operate reliably over a very wide temperature range (−269 °C to 400 °C), making them attractive for sensing applications in harsh environments when properly integrated with robust substrates [28,29]. To leverage these qualities, this work aims to integrate the PI into an LTCC-based platform to implement improved mechanical detection devices through two mechanically tuned RF passive components: a planar spiral inductor and capacitor, each operating according to distinct physical tuning mechanisms. This dual implementation allows for the evaluation and direct comparison of two fundamental detection principles, magnetic-field-based inductance tuning and electrostatic air-gap modulation, within the same hybrid platform.

**Figure 1:**
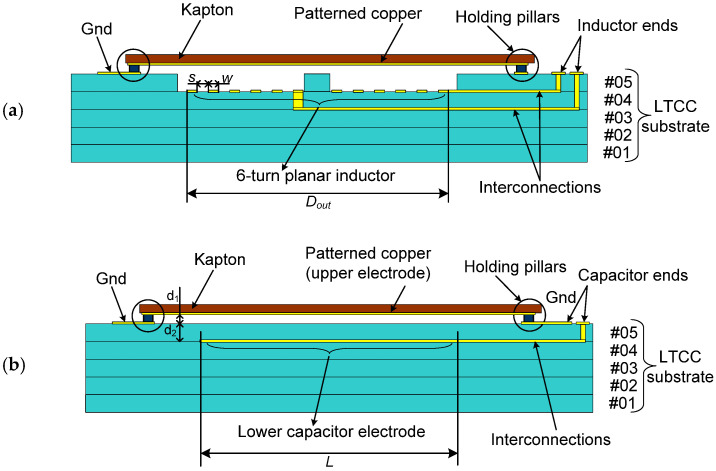
Cross-sectional schematics of the proposed hybrid LTCC–Kapton^®^ tunable passive components: (**a**) planar spiral inductor tuned via a metal-shielding proximity effects enabled by a deformable Kapton^®^ membrane, and (**b**) MIM capacitor tuned by force-controlled air-gap. LTCC layers, interconnections, and holding pillars are indicated (Dimensions are not to scale).

For the inductor, tunability is achieved using a metal-shielding tuning (MST) mechanism, a well-established technique for controlling the effective inductance of planar coils through proximity-induced magnetic field perturbation [30]. A cross-sectional schematic of the proposed hybrid LTCC–polyimide tunable planar inductor is shown in Figure 1a. The shielding element consists of a square copper plate patterned on a flexible polyimide membrane and electrically grounded. The planar spiral inductor is embedded within the LTCC rigid substrate directly beneath the movable shielding plate. When an external force is applied to the Kapton^®^ membrane, the air gap separating the inductor and the grounded metal plate is reduced. This reduced separation alters the magnetic field distribution around the spiral, inducing eddy currents in the conductive plate that generate an opposing magnetic field. As a result, the mutual inductance between the spiral and its environment is reduced, leading to a decrease in the effective inductance. To prevent electrical short-circuiting during actuation, the inductor is incorporated within the LTCC sheets to mechanically avoid any direct contact, ensuring safe operation even under large applied forces while preserving a wide inductance tuning range. To prevent electrical short-circuiting during actuation, the inductor is embedded and recessed within the LTCC stack, thereby mechanically preventing direct contact with the movable shielding plate. This design ensures reliable operation under large applied forces while maintaining a wide inductance tuning range.

Using the same hybrid substrate, capacitance tuning is achieved using an air-gap modulation mechanism, as illustrated in Figure 1b. The capacitor adopts a metal–insulator–metal (MIM) configuration, in which the lower electrode is embedded within the LTCC substrate and the upper electrode is patterned on the Kapton^®^ membrane. Mechanical deformation of the polyimide membrane under an applied force reduces the air-gap thickness between the electrodes, thereby increasing the effective capacitance via purely electrostatic coupling. The total dielectric stack consists of a variable air layer in series with a fixed-thickness LTCC ceramic layer, allowing for continuous, reversible, and geometry-controlled capacitance tuning. Together, proximity-induced magnetic shielding for inductance modulation and air-gap dielectric variation for capacitance modulation constitute the two fundamental operating principles evaluated in the proposed hybrid LTCC–Kapton^®^ high-sensitivity mechanical sensing platform.

**Figure 2:**
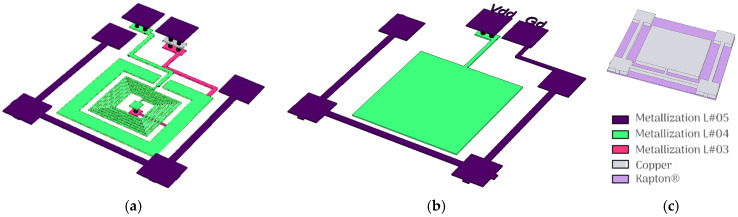
Layouts of LTCC-integrated passive components: (**a**) planar spiral inductor with underpass and via connections, (**b**) MIM capacitor lower electrode on LTCC, and (**c**) copper upper electrode patterned on the flexible Kapton^®^ membrane for tuning.

The fabrication of planar passive components using LTCC technology involves stacking multiple ceramic layers with patterned metallization levels and vertical interconnections through vias. The LTCC device developed in this work consists of five ceramic layers, labeled #L05 as the topmost and #L01 as the bottommost layer (Figure 1). The top layer (with reference DP951P2) has a reduced thickness of 6.5 mil (~165 µm) to enhance tuning efficiency, while the four underlying layers (with reference DP951PX) are each 10 mil (~254 µm) thick to provide mechanical rigidity and structural stability. Among various inductor geometries, the planar spiral topology is widely favored in RF applications due to its high inductance density, which results from positive mutual inductance between adjacent turns [30]. The implemented LTCC spiral inductor is realized as a double-layer metallization configuration, where the main multi-turn spiral is patterned on the #L04 layer, and an underpass routing line is realized on the lower metallization layer, #L03 (Figure 2a). Vertical vias connect the inner terminal of the spiral on #L04 to the underpass on #L03, enabling a compact layout without conductor crossings on the same metallization level. The spiral is screen-printed using a thick-film gold paste (25 µm thickness before co-firing, electrical conductivity 2.4 × 10^6^ S/m), with a line width (*w*) and spacing (*s*) of 100 µm to balance electrical performance and LTCC manufacturing constraints. The remaining geometric parameters are summarized in Table 1. To optimize the MST mechanism, a square cavity slightly larger than the spiral inductor is integrated into the top LTCC layer (#L05). A central cross-shaped ceramic feature is retained at the center of the cavity to act as a mechanical deflection stopper, preventing excessive membrane displacement under high applied forces (Figure 1a). This air-filled cavity, positioned directly above the spiral, enhances tunability while improving high-frequency performance by: (i) effectively eliminating the dielectric losses associated with ceramic materials, since air exhibits an extremely low loss tangent (tan δ ≈ 0), thereby reducing RF energy dissipation; (ii) significantly decreasing parasitic capacitance between the inductor and the shielding plate due to the lower permittivity of air, which helps preserve a higher self-resonant frequency (SRF) and extends the usable operating bandwidth; and (iii) minimizing unwanted capacitive coupling within the tuning gap due to reducing dielectric loading, allowing the inductance modulation to be predominantly governed by magnetic interaction with the movable shield.

On the other hand, the capacitor design is based on a metal–insulator–metal (MIM) configuration (Figure 1b and Figure 2b), where dielectric layers are sandwiched between two conductive electrodes. The lower electrode is patterned on the #L04 LTCC metallization level (the same as the spiral top layer). The upper electrode is formed by a copper plate patterned on the flexible Kapton^®^ membrane, which acts as the movable top plate. Unlike the spiral inductor, this design requires only a single metallization level, as no conductor crossings are needed. To differentiate from the inductor’s cavity-based tuning, no openings are added in any LTCC sheets. Instead, the device relies on a composite dielectric stack comprising two dielectric media connected in series between the electrodes: a variable air gap of thickness d_1_ with a relative permittivity *ε_r_*_1_ ≈ 1, and a fixed LTCC ceramic layer of thickness d_2_ with *ε_r_*_2_ ≈ 4.1. The effective capacitance is therefore governed primarily by the air-gap variation. As the Kapton^®^ membrane deflects under applied mechanical force, only *d*_1_ decreases continuously, producing a reversible increase in capacitance. The Kapton^®^ membrane layout carrying the patterned copper electrode, which acts as the tunable capacitor plate (or as the shielding element in the inductive configuration), is illustrated in Figure 2c.

## 3. Fabrication Process Flow

**Figure 3:**
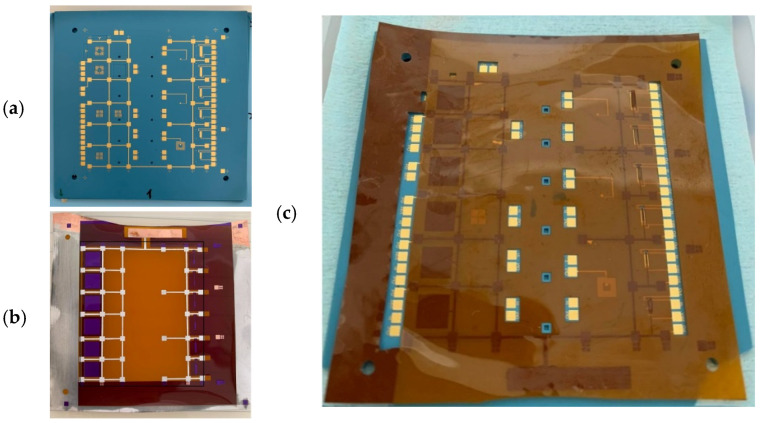
Main fabrication steps of the hybrid Kapton^®^/LTCC device: (**a**) LTCC substrate with patterned metallization and complementary holding pillar pads prior to bonding; (**b**) Kapton^®^ membrane after copper patterning (clear orange) and selective indium electroplating on holding pillars (white), designed to match the LTCC pillars; and (**c**) final assembly after low-temperature reflow bonding of the Kapton^®^ membrane onto the LTCC substrate (The overall LTCC substrate size is 8 cm × 8 cm).

The mechanically tunable planar inductor and capacitor were fabricated to evaluate the feasibility and sensitivity of the proposed hybrid Kapton^®^/LTCC-based approach. The process relies on low-temperature bonding of a flexible Kapton^®^ membrane onto a rigid LTCC substrate using selectively electroplated indium holding pillars, which simultaneously define the air gap, provide mechanical fixation, and ensure electrical grounding of the movable copper structures. Figure 3 illustrates the main fabrication steps, including LTCC platform preparation with embedded passive compounds, Kapton^®^ membrane patterning and indium electroplating, and final reflow-bonded assembly. The fabrication begins with the LTCC substrate, constructed from five layers of DuPont™ 951 green tapes. Via holes with a diameter of 250 µm were formed in the unfired ceramic tapes using a PM30 automatic mechanical punching machine (KMS Automation) and subsequently filled with thick-film gold paste 5738 (DuPont). Au-based thick-film (QR150 paste) lateral metallization patterns were screen-printed on selected layers to define the spiral inductor (#L04), lower capacitor electrode (#L04), holding pillars pads/walls (#L05), and routing lines (#L04 and #L03), using a 400-mesh stainless-steel screen with 15 µm emulsion thickness. To improve tunability for the inductive structure, the top LTCC layer (#L05) was laser-ablated using a MicroSTRUCT UV picosecond system (3D Micromac), whereas no cavities were required for the capacitive device. Carbon foils with matching cavity dimensions were placed in the openings to maintain stable geometries during lamination and sintering. After individual layer processing, the LTCC stack was aligned and subjected to a uniaxial pre-lamination at 70 °C under a force of 20 kN for 2 min, followed by isostatic lamination at 210 bar and 70 °C for 10 min to ensure uniform bonding and structural stability. The laminated stack was then co-fired using the standard free-sintering profile recommended for DuPont™ 951, under air atmosphere, resulting in a monolithic ceramic substrate with embedded passive components and defined cavities (Figure 3a). To compensate for LTCC shrinkage during firing, the layout was beforehand enlarged by 13.1% in the x–y directions.

In parallel, a 50 µm-thick copper-clad Kapton^®^ foil was processed to form the flexible membrane. The 50 µm copper layer was patterned via photolithography to define (i) movable upper electrodes for capacitive tuning and the inductive shielding plate; and (ii) copper bonding pads and routing, which correspond to the locations of the holding pillars on the LTCC substrate (Figure 3a). Initially, a 35 µm-thick negative dry photoresist was applied, exposed through a dedicated mask, and developed in a sodium carbonate solution (300 g Na_2_CO_3_ with 10 mL defoamer) at 28 °C for 3 min. This process selectively protected the copper regions above shielding plates and holding pillars, ensuring these exposed areas remained for subsequent steps. The unexposed copper was then chemically etched using a sodium thiosulfate etchant (850 g Na_2_S_2_O_3_ with 20 mL H_3_PO_4_) at 50 °C for 7 min, defining the final copper pattern. After etching, the remaining photoresist was removed with a commercial resist remover (SurfaceStrip 419) at 45 °C for 4 min, leaving clean and well-defined copper bonding pads and shielding structures.

To protect the copper shielding plate and upper capacitance electrodes during indium electroplating, a second dry-film photoresist layer was selectively applied, patterned using a different mask, and then developed so that only the holding pillar pads remain exposed, while the shielding plate and upper electrodes are fully covered. This selective masking ensured that indium electroplating occurred solely on the exposed copper bonding pads. After copper etching, the PI membrane recovered its mechanical thickness and compliance, while the electroplated indium pillars provide precise standoff height, mechanical fixation, and electrical grounding during the subsequent reflow bonding step. The copper shielding plate remains thin, maintaining sufficient clearance from the LTCC surface without compromising the Kapton^®^ membrane flexibility. Indium electroplating was carried out exclusively on the exposed copper pads using an NB Semiplate In 100 electrolyte, with high-purity indium (99.99%) ingot as the anode and the Kapton^®^ foil as the cathode. The process was conducted in an ultrasonic bath at a current density of 20 mA cm^−2^ and 40 °C, resulting in well-defined indium pillars grown on copper with heights controlled by plating duration (Figure 3b). Notably, the copper shielding plate remained unplated, preserving its original thickness; this ensured sufficient separation from the LTCC surface and maintained the mechanical compliance of the membrane. For proper mating during reflow, the indium-coated pads on the Kapton^®^ membrane were precisely aligned with the complementary pillar features on the LTCC substrate, such that each opposing pair formed a reliable mechanical and electrical connections. Consequently, the electroplated indium pillars both defined the air gap and served as localized bonding interfaces. Finally, pad openings through the Kapton^®^ foil for electrical probing were defined via laser cutting using a 3DMM MicroStruct C system. The patterned Kapton^®^ membrane was then aligned to the LTCC substrate under an optical microscope, ensuring accurate registration between the indium-coated pillars on the Kapton^®^ side and the corresponding gold-metallized pads on the LTCC substrate.

Bonding was achieved using a low-temperature reflow soldering process in a vacuum soldering oven, reaching a peak temperature of approximately 170 °C for 1 min. During reflow, the indium pillars, characterized by a low melting temperature of approximately 156 °C, melted and wetted the opposing copper and gold surfaces, forming robust mechanical and electrical interconnections upon solidification (Figure 3c) [31]. Notably, only the indium pillars participated in the soldering process, while the copper shielding plate remained solid and mechanically independent, thereby preserving the flexibility of the membrane. The process was specifically designed to minimize thermal exposure, generally keeping the assembly below 170 °C to prevent polymer degradation and reduce thermomechanical stress. Particular attention was given to the mismatch in coefficients of thermal expansion (CTE) between the Kapton^®^ (30–60 × 10^−6^ K^−1^) and the LTCC (~10 × 10^−6^ K^−1^). For a temperature excursion of 150 °C, the resulting differential expansion was estimated to be approximately 90 µm over a 10 mm length. To limit this thermally induced strain and misalignment, the lateral dimensions of the PI sheet were minimized, this allowed the alignment marks to shift only slightly during the heating cycle without compromising the overall alignment. As an alternative to indium-based reflow bonding, reactive bonding using nanoscale multilayer systems [32] (e.g., Ni/Al, Al/Ti, or Ti/a-Si), deposited by sputtering or electroplating, could be considered to enable room-temperature bonding, thereby bypassing thermal mismatch issues entirely. The successful realization of this hybrid structure requires careful control of several critical parameters, including: (i) precise definition of the air-gap height between the Kapton^®^ membrane and the LTCC surface, (ii) appropriate sizing of the copper shielding plate to ensure flatness and uniform mechanical response, and (iii) wrinkle-free bonding of the polyimide film. Notably, because no buried cavities or deformable ceramic membranes are employed, the proposed design inherently eliminates the risk of LTCC warpage or curling after co-firing.

## 4. Inspection and Electrical Parameter Extraction

**Figure 4:**
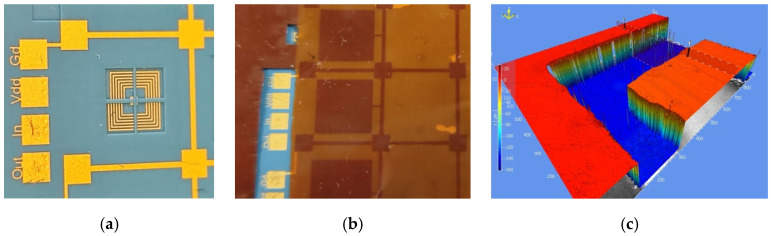
Top-views: (**a**) the LTCC planar spiral inductor with contact pads and holding pillars prior to Kapton^®^ bonding; (**b**) after bonding of the Kapton^®^ membrane carrying the grounded copper shielding plate and indium-plated pillars; and (**c**) three-dimensional surface profile of the final hybrid structure measured with the vibrometer, highlighting the separation between the Kapton^®^ membrane (red) and the LTCC substrate (blue).

The top view of the planar spiral inductor fabricated on the LTCC substrate is shown in Figure 4a. The spiral is surrounded by a grounded shielding ring that also forms the mechanical holding pillars supporting the suspended polyimide (PI) membrane. These pillars define the lateral dimension of the membrane (10.6 mm × 9.8 mm for the capacitance) and are electrically connected to ground through the dedicated ‘Gd’ pad, as indicated in Figure 4a. A cross-shaped opening is laser-cut in the top LTCC layer (#L05), exposing the spiral region and simultaneously acting as a mechanical stopper to limit membrane deflection. After bonding the Kapton^®^ membrane, the inductor is entirely covered by a grounded copper shielding plate patterned on the flexible polyimide film (Figure 4b). To verify the fabricated geometry and the flatness of the membrane, a three-dimensional surface scan of the hybrid structure was performed using a Polytec vibrometer. The resulting topographical map provides the height profile of both the recessed LTCC region and the suspended membrane. Special attention was given to the air gap between the LTCC surface and the Kapton^®^ membrane, since this distance determines the available deflection range and thus strongly affects the tuning behavior. As illustrated in Figure 4c, the 3D scan is taken along both the upper surface of the Kapton^®^ membrane and the recessed groove that defines the top surface of the LTCC substrate; from these two profiles, a mean vertical separation of about 140 µm was obtained. Considering the 50 µm Kapton^®^ film and the 50 µm copper layer, the initial air gap between the bottom surface of the copper shielding plate and the LTCC surface is therefore approximately 40 µm.

**Figure 5:**
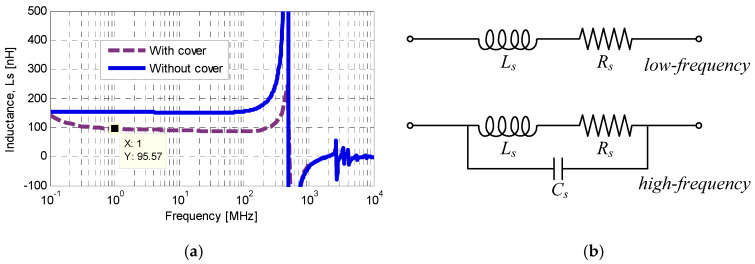
The LTCC planar spiral inductor: (**a**) self-inductance versus frequency with and without a copper shielding plate; (**b**) low-frequency and high-frequency lumped-element equivalent circuits.

Once the geometrical parameters were determined, the passive component characteristics were extracted numerically using Advanced Design System (ADS). Figure 5a reports the simulated self-inductance of the spiral inductor as a function of frequency, comparing the unshielded configuration with the case where a copper plate located 40 µm above the inductor. The self-inductance (*L_s_*) and series resistance (*R_s_*) were extracted from the admittance matrix (Y-matrix) using the *Y*_11_ parameter according to [33]:

(1) $$L_{s}=\frac{I\left[{\left(Y_{11}\right)}^{-1}\right]}{\omega }$$

(2) $$R_{s}=R\left[{\left(Y_{11}\right)}^{-1}\right]$$

where ω denotes the angular frequency. As observed in Figure 5a, at low frequency the inductance remains nearly constant at 154 nH in the absence of shielding. When the copper plate is present at *d*_2_, the low frequency inductance decreases to about 95 nH. As the frequency approaches the SRF, around 470 MHz, the inductance deviates from this constant value because of increasing capacitive coupling. The inductor behavior can be described by the lumped element equivalent circuits shown in Figure 5b. At low frequencies, the model consists of an ideal inductance (*L_s_*) in series with a resistance (*R_s_*), which accounts for conductive and frequency-dependent losses. At higher frequencies, an additional parasitic inter turn capacitance (*C_s_*) gives rise to the SRF. Well below resonance, these elements can be treated as frequency independent and the simple series *L_s_*–*R_s_* model accurately reproduces the inductor response.

**Figure 6:**
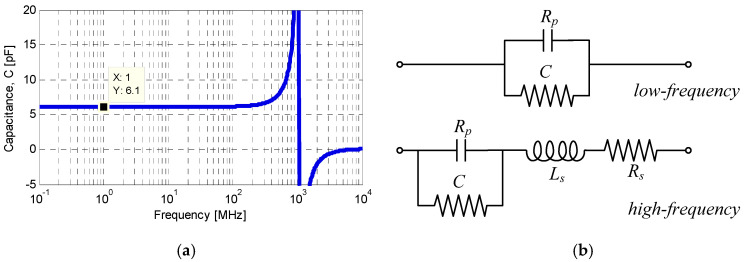
The MIM thick-film capacitor: (**a**) capacitance versus frequency; (**b**) low-frequency and high-frequency lumped-element equivalent circuits.

A similar modelling procedure was applied to the MIM capacitor in ADS, using the geometrical dimensions obtained from the surface-topography measurements. The frequency-dependent capacitance extracted from the Y matrix via Equation (3), is plotted in Figure 6a and is approximately 6.1 pF at low frequencies. The insulation resistance was extracted from the same Y matrix using Equation (4), yielding the parallel resistance in the equivalent circuit.

(3) $$C=\frac{I\left[Y_{11}\right]}{\omega }$$

(4) $$R_{p}=\frac{1}{R\left[Y_{11}\right]}$$

The theoretical capacitance of the structure can be estimated using the parallel-plate expression, *C* = *ε A*/*d*, where *ε* is the absolute permittivity, A the electrode overlap area, and *d* is the dielectric thickness [34]. In this device, the dielectric stack consists of a variable air gap of thickness *d*_1_ and relative permittivity *ε_r_*_1_ ≈ 1 in series with a fixed LTCC ceramic layer of thickness *d*_2_ and relative permittivity *ε_r_*_2_ ≈ 4.1; the resulting effective capacitance is therefore given by:

(5) $$C_{eff}=\;\frac{\varepsilon_{0}L^{2}}{\frac{d_{1}}{\varepsilon_{r_{1}}}+\frac{d_{2}}{\varepsilon_{r_{2}}}}$$

It should be noted that the in-plane (X/Y) shrinkage of 12.7% occurring during LTCC co-firing was compensated at the layout stage by proportionally enlarging the initial dimensions. However, the vertical (Z-axis) shrinkage of approximately 12–15% is unavoidable and results in a reduction of the fired LTCC layer thickness as well as the thickness of the metallization lines, which decreases to approximately 8–9 µm after sintering. Assuming a uniform electric field—which is justified because the electrode area is much larger than the total dielectric thickness—and using the measured values (d_1_ = 40 µm, d_2_ = 140 µm, and electrode side length L = 7 mm), the calculated capacitance is 5.86 pF. This agrees well with the 6.1 pF obtained from full-wave electromagnetic simulation. Accordingly, the MIM capacitor can be represented by the equivalent circuits shown in Figure 6b. At low frequencies, a parallel RC model accurately describes the insulation resistance and storage capacity. At higher frequencies, the model is augmented to include parasitic effects arising from the leads and metallization.

## 5. Tuning Mechanism and Underlying Physics

**Figure 7:**
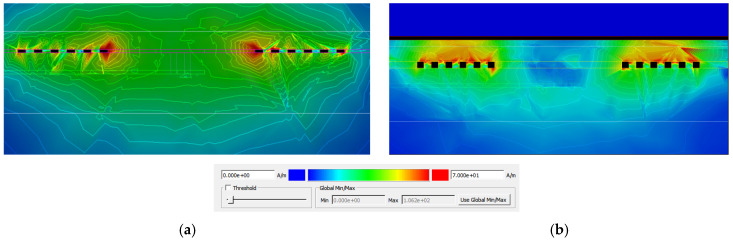
Simulated magnetic field strength distribution of the LTCC planar spiral inductor: (**a**) without proximity shielding; (**b**) with a grounded copper shielding plate, illustrating magnetic field attenuation due to eddy-current effects (red regions indicate higher field intensity).

The tuning of the LTCC-integrated spiral inductor relies on a metal shielding tuning (MST) concept that exploits the controlled proximity of a conductive plate to the inductor. The shield element is a grounded 7 × 7 mm^2^ copper plate, 50 µm thick, patterned on the Kapton^®^ membrane and characterized by a conductivity of *σ*_Cu_ = 6.2 × 10^7^ S·m^−1^ and a relative magnetic permeability slightly below unity (*µ_r_* = 0.999994), typical of non-magnetic conductors. Figure 7 illustrates the ADS-simulated magnetic field strength (H) distribution around the inductor. In the absence of proximity shielding, the magnetic field is concentrated near the central region of the spiral, with flux lines extending freely into the surrounding medium (Figure 7a). When the grounded copper plate is brought above the inductor, a significant attenuation of the magnetic field strength is observed in the region between the inductor and the shield (Figure 7b). This behavior arises from the interaction between the time-varying magnetic field generated by the inductor and the conductive shielding plate. As the copper plate approaches the inductor, a portion of the magnetic energy is coupled into the conductive material, inducing circulating currents known as eddy currents. According to Lenz’s law, these eddy currents generate a secondary magnetic field that opposes the original field of the inductor. This counteracting magnetic flux reduces the net magnetic flux linkage (φ) across the spiral turns, thereby decreasing the effective inductance, as defined by L = φ/I. This proximity-induced magnetic shielding is the fundamental physical mechanism governing the inductance tuning.

**Figure 8:**
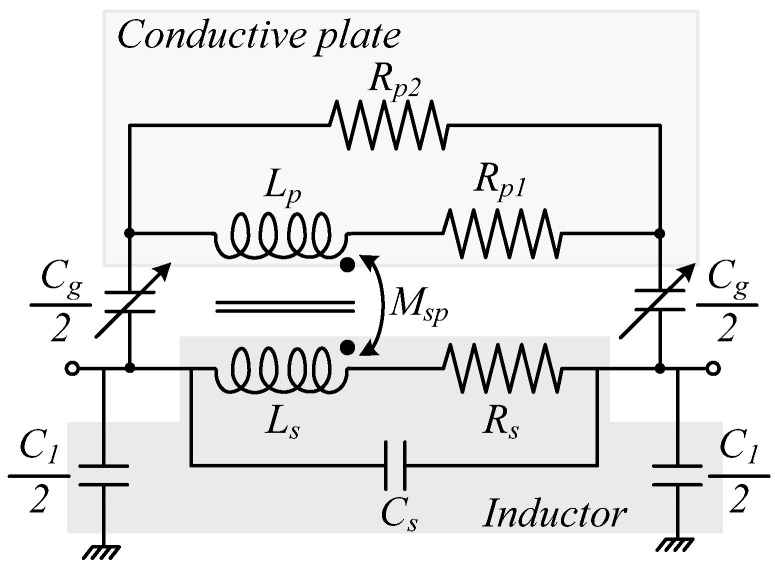
Lumped-element equivalent circuit model of the mechanically tunable LTCC planar spiral inductor with a grounded proximity shielding plate, including parasitic capacitances and magnetic coupling effects [30].

A quantitative description of the MST effect is based on the two-port lumped-element model shown in Figure 8. While the intrinsic parameters of the spiral (L_s_, R_s_, C_s_) are determined by the LTCC geometry, the tuning behavior is governed by the interaction with the external shielding plate. This plate is modelled as an equivalent LR network (L_p_, R_p1_, R_p2_) located on the secondary side of a magnetically coupled transformer. The inductance *L_p_* models the effective loop inductance of the eddy-current paths induced in the plate by the time-varying field of the spiral. The resistance *R_p_*_1_ accounts for ohmic losses associated with these eddy currents, which depend on the plate conductivity, thickness, and frequency-dependent skin-depth. The additional resistor *R*_*p*2_ represents further distributed loss mechanisms in the shield, such as non-ideal current distribution, finite contact resistance to ground, and possible substrate loss under the plate; it is often introduced to fit the measured or simulated quality factor of the tuned inductor more accurately. The core of the tuning mechanism lies in the magnetic coupling between the spiral and the shield, characterized by the mutual inductance *M_sp_*, and the coupling coefficient *k_sp_*, defined as [30]:

(6) $$k_{sp}=\frac{M_{sp}}{\sqrt{L_{s}L_{p}}}$$

Physically, *M_sp_* measures how much of the magnetic flux generated by the current in the spiral links the eddy-current loops in the plate (and vice versa), while *k_sp_* is a normalized version of this coupling that satisfies 0 ≤ *k_sp_* ≤ 1. As the copper plate moves closer to the inductor, the spatial overlap between the magnetic field of the spiral and the conductive area increases, so that both *M_sp_* and *k_sp_* grow in magnitude, reinforcing the shielding effect and hence the reduction of the effective inductance observed in Figure 5. In addition to magnetic coupling, the model includes the capacitance *C_g_* between the spiral tracks and the shielding plate, as well as the capacitances *C*_1_ between the inductor tracks to the surrounding ground metallization. These elements originate from the finite area overlap between the metal conductors and the grounded structures, separated by the LTCC dielectric and, in the case of *C_g_*, by the air gap. As the plate approaches the spiral, the distance *g* between the conductors decreases, so *C_g_* increases approximately as 1/g. This not only raises the total parasitic capacitance seen by the inductor but also lowers the SRF. In contrast, *C*_1_ is primarily determined by the LTCC stack-up and the ground layout, and is therefore only weakly affected by the plate displacement [30]. Among all parameters, *M_sp_*, *k_sp_*, and *C_g_* are the most strongly dependent on the vertical separation *g* between the spiral and the shield, because they are directly controlled by the spatial overlap and distance between the field lines of the inductor and the conductive plate. As *g* is reduced, the increased coupling (larger *k_sp_* and *M_sp_*) and larger *C_g_* jointly explain the pronounced decrease of the low-frequency inductance and the shift of the SRF seen in Figure 5.

At frequencies well below the SRF, the impedances of all capacitive elements C_s_, C_g_, and C_1_ are much larger than the inductive reactances, so these capacitances can be considered open circuits. Hence, the equivalent circuit reduces to (i) a primary branch containing Ls and Rs in series (the LTCC spiral); and (ii) a magnetically coupled secondary branch containing L_p_ and R_p1_–R_p2_ in series (eddy-current loops in the plate), linked to the primary via the mutual inductance M_sp_. Electrically, this is equivalent to a single inductor whose effective inductance L_eff_(g) is lower than L_s_ because the shield behaves as a short-circuited secondary winding that partially cancels the flux of the primary. As the plate approaches the spiral (smaller g), the coupling *k_sp_* increases, more flux is diverted into eddy-current loops, and the reflected impedance of the shield reduces *L_eff_*(g) from 154 nH (no plate) down to about 95 nH at g ≈ 40 µm, in agreement with the results shown in Figure 5. This simple low-frequency transformer scenario is therefore sufficient to interpret the measured tuning curves without invoking capacitances, which only become important when the operating frequency approaches the SRF. It is worth mentioning that at low frequencies the eddy currents remain relatively weak, so the magnetic shielding is less pronounced and the resulting inductance variation with plate position is significantly smaller.

While the MST principle is applied to tune the inductance, the capacitor operates through a different mechanism. In the hybrid MIM capacitor implemented in the LTCC/Kapton^®^ stack, the capacitance is adjusted by changing the distance between the LTCC-integrated bottom electrode and the movable grounded top plate. Varying this air gap directly modifies the electrode spacing and the electric-field distribution, which changes the effective capacitance C, while the dielectric properties of the LTCC and the Kapton^®^ remain constant. This approach allows precise adjustment of the low-frequency capacitance without altering the lithographically defined geometry of the embedded LTCC electrode.

## 6. Experimental Results and Comparisons

**Figure 9:**
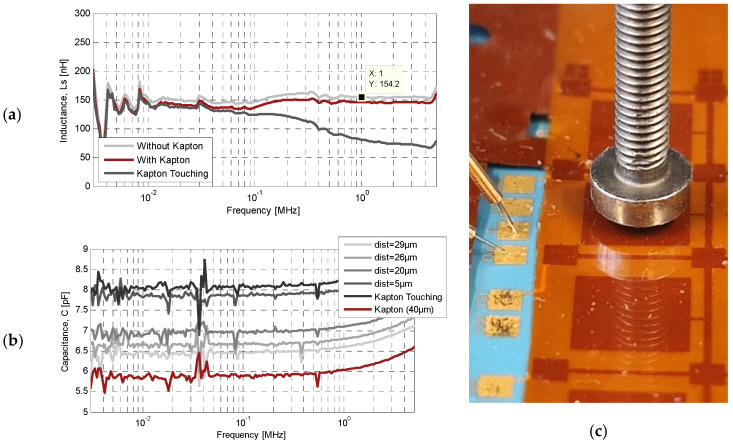
Spiral inductor and capacitor measurements: (**a**) measured inductance versus frequency for different shield positions, (**b**) measured capacitance versus frequency for different electrode separation gaps, and (**c**) photograph of the experimental setup showing probe needles and the mechanical loading tip with a diameter of 5 mm.

The RF characterization of the fabricated devices was carried out with an ST2839 impedance analyzer (LCR meter series) operating over a frequency range of 20 Hz to 10 MHz, using a probe station with two sharp probe needles to ensure stable contact to the LTCC pads. Open and short calibrations were performed before the measurement sequence in order to remove the parasitic impedance of the probes and cables at all selected frequency points. For the inductor, the complex impedance Z_11_ was measured from 1 kHz to 5 MHz, and the equivalent lumped parameters were extracted from the real and imaginary parts of Z_11_. The measured self-inductance versus frequency is shown in Figure 9a. At 1 MHz, the measured inductance is 154.2 nH, which is in excellent agreement with the simulated low-frequency value of 154 nH, with a series resistance of 8.57 Ω. The slight deviation is primarily attributed to the ±5% tolerance in line width from the LTCC screen printing process. To evaluate the tunability, the inductor was subjected to quasi-static out-of-plane actuation using a TA Instruments DMA2980 dynamic mechanical analyzer operated in force-controlled mode. A flat-ended screw loading tip aligned over the center of the Kapton^®^ membrane applied a calibrated normal force along the z-axis (Figure 9c), while the DMA2980 simultaneously recorded the applied load and the resulting membrane deflection. At each incremental force step, the electrical response of the inductor was measured with the ST2839 impedance analyzer, tracking the evolution of the inductance as the air gap progressively decreased. The onset of mechanical contact between the tip and membrane was identified by a reproducible change in the low-frequency inductance, marking the start of gap reduction. Thanks to the cross-shaped opening in the top LTCC layer (#L05), a residual air pocket remains confined even at maximum deflection, preventing direct contact between the copper plate and the spiral and thus avoiding electrical shorting. Figure 9a presents the measured inductance spectra for the three key configurations. In the bonded but rest position, the inductance already exhibits a moderate reduction relative to the unshielded case, due to the finite magnetic coupling and parasitic capacitance to the grounded copper plate at the initial air gap. As the applied force increases and the plate approaches the spiral, the low-frequency inductance decreases monotonically, with the largest tuning effect observed above 200 kHz. It is worth noting that at the lowest frequencies (below ~100 kHz), the eddy currents induced in the shield remain relatively weak, so the magnetic shielding is less pronounced and the resulting inductance variation with plate position is significantly smaller. At 1 MHz and maximum deflection (separation ≈ 140 µm, equal to a single LTCC layer thickness), the inductance decreases to ~80 nH, corresponding to a tuning range of roughly 48% relative to the unshielded value. The tuning range can be further increased by enlarging the initial air gap, for example by increasing the electroplated indium pillar height [35].

The hybrid MIM capacitor was characterized using the same force-controlled setup. The air-gap separation *d*_1_ was varied from its rest position of 40 µm (undeformed membrane) down to full contact with the LTCC surface. As shown in Figure 9b, the capacitance at 1 MHz increases from 6.03 pF to 8.21 pF as the gap decreases, corresponding to a tuning range of 36%. This monotonic increase is consistent with the parallel-plate capacitor model, where the reduction of the air-gap thickness enhances the electric-field coupling between the LTCC-embedded bottom electrode and the movable copper top plate. Because the dielectric properties of the LTCC and Kapton^®^ remain unchanged, tuning is purely a function of the mechanical gap modulation. The architecture therefore preserves the simplicity of a conventional MIM capacitor while significantly extending its tuning range via compliant membrane displacement.

**Figure 10:**
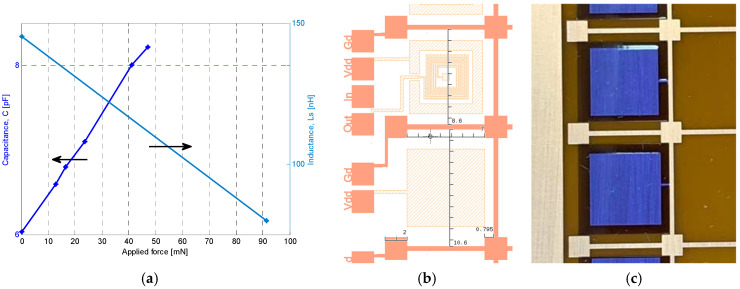
(**a**) Electromechanical tuning characteristics at 1 MHz: Inductance and capacitance versus the applied force; (**b**) device layouts showing the membrane dimensions and exact pillar geometry, and (**c**) Kapton^®^ membranes after copper patterning and selective indium electroplating (inductive shielding plate on top, capacitive electrode on bottom).

Figure 10a presents the inductance and capacitance measured at 1 MHz as a function of the applied force, illustrating both the achievable tuning range and the nearly linear electromechanical response of the hybrid platform. The Kapton^®^ membranes differ in size between the two devices: 10.6 mm × 9.8 mm for the capacitive element and 8.6 mm × 9.8 mm for the inductive element, as detailed in Figure 10b. This difference in membrane area and stiffness results in the inductive structure sustaining higher forces (up to ~100 mN), whereas the capacitive device was characterized up to ~50 mN under identical loading. Figure 10b depicts the membrane dimensions and pillar geometry in the layout, while Figure 10c shows the fabricated Kapton^®^ membranes after electroplating. Mechanical sensitivity, defined as the change in electrical parameters per unit force (ΔL/F and ΔC/F), is extracted from the slopes in Figure 10a. At 1 MHz, the average sensitivities are 0.715 nH/mN for inductance and 47.3 fF/mN for capacitance, reflecting strong electromechanical coupling afforded by the thin initial air gap and the high compliance of the Kapton^®^ membrane. The device exhibits linear and reversible deflection up to 100 mN (equivalent to <5 kPa for a 5 mm-diameter loading tip) without electrical shorting, thanks to the integrated ceramic stopper structure, ensuring mechanical robustness under overloading.

State-of-the-art LTCC-based mechanical sensors increasingly favor LC resonant architectures over simple parallel-plate capacitors due to superior practical sensitivity and measurement efficiency. LC resonant sensors convert small mechanical perturbations into measurable frequency shifts, effectively amplifying minor capacitance changes (ΔC~1–5%) into fractional frequency variations (Δf/f_0_), typically 1–10%. Several implementations highlight this advantage, for instance, Lin et al. [7] demonstrated an LTCC sensor with a 6.5 mm × 6.5 mm × 395 μm cavity, achieving 3.76 kHz/kPa for membranes of 148 μm thickness, while thicker membranes (432 μm) extended the detection range up to 2660 kPa. Gao et al. [36] implemented an HTCC sensor using a 100 μm high cavity with a 4 mm radius plate, reporting 38 kHz/bar sensitivity over a 60-bar range at temperatures up to 650 °C. Qin et al. [37] employed DuPont 951 LTCC with a 9 mm cavity, obtaining 1.5 kHz/bar at 20 °C and demonstrating linear response up to 400 °C. Tan et al. [38] used LTCC interdigital capacitors with evacuation cavities, achieving 274 kHz/bar up to 2 bar and wireless readout at 600 °C. Ma et al. [39] presented a planar spiral inductor (20 mm outer, 11.5 mm inner, 6 turns) paired with an interdigital capacitor, where proximity-induced eddy currents modulated the resonant frequency with 11.2 MHz/mm sensitivity over 1–3 mm distances. These works highlight that LC architectures are particularly suited for wireless operation, high-temperature stability, and high signal-to-noise ratio, whereas purely capacitive approaches remain limited to wired, high-resolution laboratory measurements. Common geometries include planar spiral inductors combined with cavity or interdigital capacitors, where mechanical pressure or proximity modulates membrane deflection or induces eddy currents. In comparison, the proposed LTCC/Kapton^®^ hybrid platform experimentally validates a proof-of-concept for high-sensitivity mechanical sensing without requiring deformable ceramic membranes or buried cavities—a key novelty over conventional LTCC approaches. Its combination of robust LTCC substrates, flexible polyimide membranes, low-cost fabrication, and simple design enables wide-range tunable RF components suitable for harsh environments. This approach opens opportunities for integration into wearable biomedical devices, soft robotics, automotive tire-pressure systems, and low-temperature aerospace structural health monitoring.

From a materials standpoint, LTCC substrates can operate reliably above 200 °C, while Kapton^®^ polyimide foils tolerate continuous use up to ~300 °C. In the current implementation, however, the indium-based low-temperature bonding imposes a limit (~156 °C melting point), making the conservative safe operating range 120–130 °C, sufficient for many industrial and automotive applications. Detailed studies on potential improvements, temperature-dependent tuning, sensitivity, and long-term drift are planned for future work.

## 7. Conclusions

This work demonstrates a high-sensitivity, mechanically tunable sensor based on an LTCC/Kapton^®^ hybrid platform. The concept is realized by bonding a flexible Kapton^®^ membrane onto the LTCC substrate using low-temperature reflow soldering, with selective indium electroplating forming supporting pillars and precise air-gap control. This architecture enables modulation of inductance through metal-shielding proximity effects and tuning of capacitance via membrane deflection, combining high mechanical compliance with structural stability. Integrating the compliant membrane with multilayer LTCC allows precise electromechanical tuning while maintaining robust electrical performance and eliminating the need for deformable ceramic membranes or buried cavities. Experimental results confirm a 48% inductance tuning range with 0.715 nH/mN sensitivity and a 36% capacitance tuning range with 47.3 fF/mN sensitivity, with strong linear electromechanical coupling. The inclusion of a ceramic stopper ensures safe operation under high forces. This hybrid approach provides a scalable route for mechanically reconfigurable RF components and high-sensitivity sensors for harsh environments.

## Figures and Tables

**Table 1:** Geometrical dimensions of the LTCC-based inductor and capacitor shown in Figure 1.

	Inductor				Capacitor		
Parameter	*n*	*D_out_* [mm]	*w* [µm]	*s* [µm]	*L* [mm]	*d*_1_	*d*_2_ [mils]
Value	6	4	100	100	7	variable	6.5

## Data Availability

Dataset available on request from the authors.
